# Hemodynamic Mechanism of Coronary Artery Aneurysm High Occurrence on Right Coronary Artery

**DOI:** 10.3389/fphys.2020.00323

**Published:** 2020-04-16

**Authors:** Dandan Wu, Sirui Wang, Jinsheng Xie, Boyan Mao, Bao Li, Chunbo Jin, Yue Feng, Gaoyang Li, Youjun Liu

**Affiliations:** ^1^College of Life Science and Bioengineering, Beijing University of Technology, Beijing, China; ^2^Beijing Anzhen Hospital, Capital Medical University, Beijing, China

**Keywords:** coronary artery aneurysm, high occurrence on right coronary artery, geometric multi-scale model, fluid-structure interaction, creep simulation

## Abstract

The abnormal diameter of the coronary artery is twice or more than the normal diameter, which is a coronary artery aneurysm (CAA). According to the clinical statistics, CAA shows high occurrence on right coronary artery (RCA). The most common cause of CAA in adults is atherosclerosis, which destroys the elastic fibers in the middle layer of the blood vessel. Under the intravascular pressure, the weak wall bulges outward and form CAA. This article aims to explain the hemodynamic mechanism of coronary artery aneurysm shows high occurrence on RCA. Occurrence of CAA was simulated by the volume growth of coronary artery. Firstly, a 0–3D multi-scale model of normal coronary artery was constructed to obtain the hemodynamic environments of coronary artery. Then, fluid-structure interaction of normal and atherosclerotic blood vessel was performed to obtain volume growth rate of the coronary artery. Atherosclerosis was simulated by modifying Young’s modulus in middle layer of the blood vessel. Finally, creep simulation was performed to compare the deformation of the blood vessels under the accumulation of time. Under normal condition, the volume growth rate of the RCA is 2.28 times and 1.55 times of the LAD and the LCX. After atherosclerosis, the volume growth rate of the RCA was 2.69 times and 2.12 times of the LAD and the LCX. And the volume growth rate of the RCA was 3.85 times and 3.45 times of the LAD and the LCX after further deepening of atherosclerosis. The expansion time above the average volume growth rate of the RCA, the LAD and the LCX respectively were 0.194, 0.168 and 0.179 s. The RCA is 2.06 times the original, the LAD and LCX are 1.53 times and 1.56 times after 10 years in creep simulation. It can be concluded that the RCA is more prone to aneurysms originated from the larger expansion of the RCA under normal physiological condition, and the larger expansion is magnified under atherosclerosis condition with destroyed vessel elasticity, and further magnified during the time accumulated viscoelastic creep to develop to aneurysm eventually.

## Introduction

The abnormal diameter of the coronary artery is twice or more than the normal diameter, which is a coronary aneurysm (Syed, 1997; Pahlavan and Niroomand, 2006; Jariwala et al., 2018) (Coronary Artery Aneurysm, CAA).

The most common cause of CAA in adults is atherosclerosis, which destroys the elastic fibers in the middle layer of the blood vessel. Under the intravascular pressure, weak wall bulges outward and forms CAA (Anabtawi and De Leon, 1974; Demopoulos et al., 1997; Baman et al., 2004). Pan et al. (2014) detected 697 coronary artery ectasia (CAE) patients. There were totally 878 dilated coronary arteries, and the main branch of right coronary artery (RCA) was mostly involved (41.80%), followed by the main branch of left anterior descending artery (LAD) (25.40%), the main branch of left circumflex artery (LCX) (21.64%), and the left main coronary artery (LM) (4.78%). It can be seen that the coronary aneurysm presents high occurrence on RCA. But due to the rareness of coronary aneurysm, there are only a few reports of clinical cases and their treatment (Swaye et al., 1983; Dahhan, 2015). The hemodynamic mechanism of CAA is not sufficient. So why does coronary aneurysm present high occurrence on right coronary artery?

The formation of CAA is a complex physiological process. The occurrence of CAA in adults is mainly due to atherosclerosis involving the middle layer of blood vessels. Therefore, three-layer blood vessels were established and the young’s modulus of the middle blood vessels were modified to simulate atherosclerosis. Two aspects of simulation were performed to explain that the RCA prone to form CAA. Firstly, the deformation of vessel wall can be directly reflected by Fluid-structure Interaction numerical simulation. By comparing the volume growth rate of the RCA, the LAD and the LCX in normal condition and atherosclerosis, whether RCA prone to expand could be judged. Secondly, the formation of coronary artery aneurysm is cumulative. Creep numerical simulation was performed to predict whether RCA is prone to form CAA.

## Materials and Methods

Firstly, a 0–3D geometric multi-scale model of normal coronary artery was constructed to obtain the hemodynamic environments of the RCA, LAD, and LCX. Then, fluid-structure interaction was performed under the normal condition and the atherosclerosis. Atherosclerosis was simulated by modifying Young’s modulus in middle layer of the blood vessel. Finally, creep simulation was performed to simulate the formation of CAA.

### Multi-Scale Numerical Simulation

**FIGURE 1:**
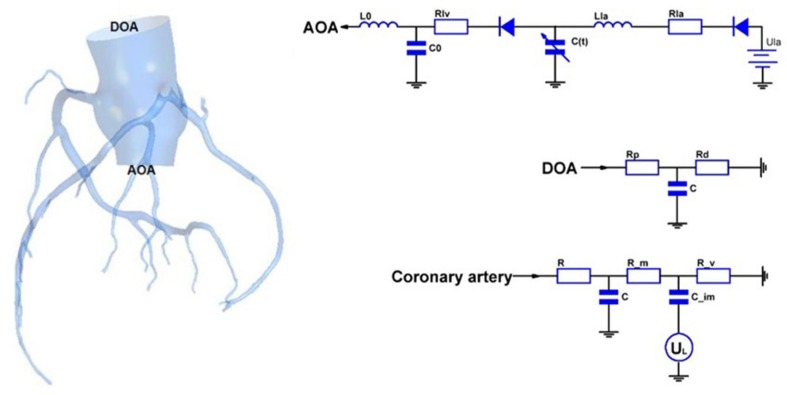
Geometric multi-scale model of normal coronary arteries.

In this study, a geometric multi-scale method (Zhao et al., 2014) is used to couple the 0D lumped parameter model with the three-dimensional coronary artery model for fluid dynamics simulation., The CT image of the coronary artery were imported to Mimics software and the 3D model reconstruction was performed by using the threshold segmentation method and manual segmentation method, respectively. Coronary artery models were eventually reconstructed in FREEFORM software based on the research objectives combined with previous actual case reports. The 0D lumped parameter model refers to the findings of Taylor et al. (2013) and has been used many times in our previous studies, and its accuracy has been confirmed (Stergiopulos et al., 1996; Zhao et al., 2016). The lumped parameter model in this study can be divided into three modules: heart module, aorta module and coronary artery module. See the right side of Figure 1. The parameter values of each component were based on the research of Kim et al. on coronary artery modeling (Kim et al., 2010), and the values were adjusted by genetic algorithm (Li et al., 2018). The systolic and diastolic pressure, and cardiac output of the model were adjusted to match the physiological reality of the patient, and made the flow of each branch of coronary artery conform to the principles proposed by Kim: The total coronary arterial flow accounts for 4% of the patient’s cardiac output. And then, the flow of each branch of the coronary artery is was then proportional to the cubic of the diameter of the branch (Kim et al., 2010). Values of 0D lumped parameters are show in Table 1.

**TABLE 1:** 0D part parameter value.

Heart module	Branch	R_la_	R_lv_	L_la_	L_1_	C_1_
	
		0.00375	0.0075	0.000285	0.0037	0.95
**Aorta module**	**Branch**	**R_p_**	**R_d_**	**C_doa_**	**C_doa_a_**	
	
		0.10285	0.9196	2.2653	0.0005	

**Coronary artery module**	**Branch**	**R**	**R_m_**	**R_v_**	**C**	**C_im_**

	a	78.6	127.52	44.51	0.00124	0.01269
	b	95.6	155.52	44.51	0.00124	0.01269
	c	83.52	136.28	42.01	0.00124	0.01269
	d	110.6	180.52	44.51	0.00124	0.01269
	e	95.6	155.52	44.51	0.00137	0.00909
	f	95.6	155.52	44.51	0.00137	0.00909
	g	79.26	129.51	65	0.00124	0.01269
	h	100.26	163.51	65	0.00124	0.01269
	i	79.26	129.51	40	0.00124	0.01269
	j	79.26	129.51	40	0.00137	0.00909
	k	79	129	60	0.00124	0.01269
	l	131.25	135.51	60	0.00137	0.00909
	m	131.25	135.51	70	0.00137	0.00909
	n	130	212	88.75	0.00124	0.01269
	o	130	212	60	0.00137	0.00909
	p	150	244	60.15	0.0001	0.0354
	q	175.5	283	60	0.0001	0.0354
	r	155.5	244	40	0.0001	0.0354

*The unit of resistance R is mmHg⋅s/mL, and the unit of blood flow inertia L is mmHg⋅s^2^/mL, the unit of vascular compliance C is mL/mmHg.*

As shown in Figure 1, the 3D model is coupled with the entrance and exit of the 0D model to form a geometric multi-scale model. The 3D model was meshed by the CFX module in ANSYS, and the meshing method was a hexahedral mesh. At the same time, it is assumed in the simulation that the blood vessel is a rigid wall that is impermeable, and the blood is an incompressible Newtonian fluid. The dynamic viscosity of blood is 0.0035 Pa⋅s and the density of blood is 1060 kg/m^3^, which is an unsteady laminar flow and calculated according to the existing method (Zhao et al., 2014). The hemodynamic parameters of the coronary arteries are finally obtained. These hemodynamic parameters, such as flow and pressure, were applied to the fluid-structure interaction numerical simulation as boundary conditions.

### Two-Way Fluid-Structure Interaction

CAA occurs mostly in trunk of coronary artery and less frequently in branches. In the objective-specific 3D coronary model, the models for the RCA, LAD, and LCX were built, as shown in Figure 1. Since LM is relatively short and the incidence of CAA is extremely low, the model of LM was not built.

**FIGURE 2:**
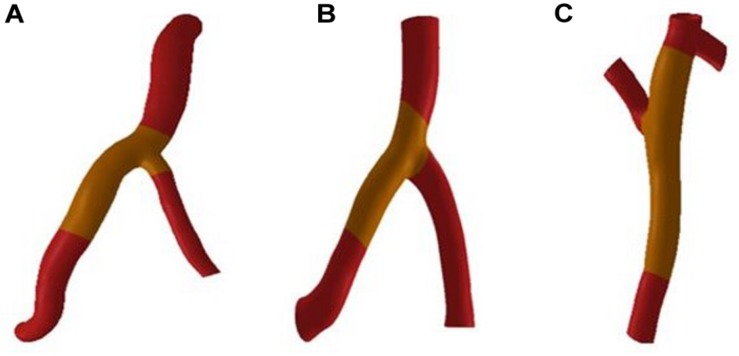
Blood vessel models. **(A)** RCA **(B)** LAD **(C)** LCX.

The fluid-structure interaction model is divided into a blood vessel model and a fluid model. First, the blood vessels shown in Figure 2 were thickened by GEOMAGIC software to obtain a three-layer blood vessel model consisting of the inner layer, the middle layer and the outer layer. In clinical statistics, there was no significantly difference in the wall thickness of RCA, LAD, and LCX. The total thicknesses of the three-layer vessel models were 0.95 mm. The thickness of the inner layer and the outer layer is 0.3 mm and the thickness of the middle layer is 0.35 mm (Li and Guo, 2017). Boolean operations are then performed on the blood vessel models in SOLIDWORKS software to tailor the fluid models. After the model is established, the two-way fluid-structure interaction was performed in ANSYS WORKBENCH software. The coupling module consisted of Transient Structural module and Fluid Flow module. Material setup, meshing, and analysis setup are performed in the Transient Structural module for the blood vessel model. The structure of the three-layer blood vessel models exhibits different properties. The Young’s modulus of the inner and outer layer was about 3 times lower than the middle layer. Therefore, we referenced (Gao et al., 2006; Watanabe and Matsuzawa, 2006) and Maier et al. (2010), who reported that the ratio of Young’s modulus of the inner layer, the middle layer and the outer layer is 1/3/1, so the Young’s modulus of the inner, middle and outer layer was set to Ei = 0.9 MPa, Em = 2.7 MPa, and Eo = 0.9 MP, respectively, and the Poisson’s ratio was set to 0.45.

Mesh independency test was performed, for example, the RCA vessel model in Figure 2. The mesh sizes of the blood vessel were set to 0.3, 0.1, and 0.05 mm. Fluid-structure interactions were performed for three groups of models with different mesh sizes. The error rate of the calculation results of the three groups of models is within 5%. In order to reduce the calculation time and increase the calculation speed, the mesh size is chosen to be 0.3 mm. The mesh number of the RCA, LAD, and LCX are 167521, 84839, and 324997.

Binding constraints were imposed between the layers of blood vessels. A cylindrical coordinate system was established at the entrance and exit of the blood vessels to restrain the circumferential and axial displacement of the blood vessels, so that the blood vessels could freely expand radially. The fluid setting was then made in the Fluid Flow module. The fluid density is 1060 kg/m^3^ and the dynamic viscosity is 0.0035 Pa⋅s. The fluid boundary conditions were taken from the previous 0–3D multi-scale numerical calculations. The calculation type is transient, taking the normal cardiac cycle *T* = 0.8 s.

**FIGURE 3:**
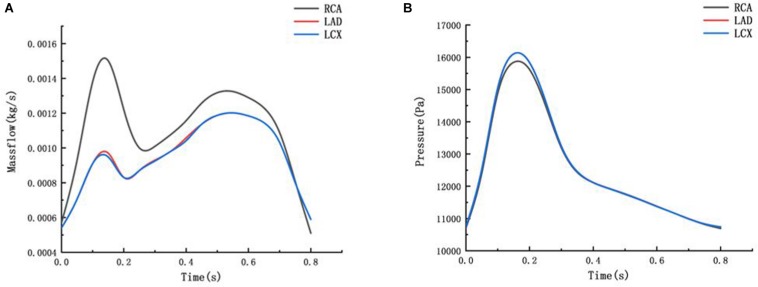
Mass flow and Pressure boundary **(A)** mass flow boundary of inlet **(B)** pressure boundary of outlet.

The flow boundary of the fluid inlet and the pressure boundary of the fluid outlet were given. The boundary settings are shown in Figure 3.

The deformation of the RCA, the LAD and the LCX under normal Young’s modulus is obtained from fluid-structure interaction. Through fluid-structure interaction simulation under normal condition, it was found that the maximum deformation of the RCA, LAD, and LCX was at the bifurcation position. The length of atherosclerosis site is 20 mm. The orange marker area in Figure 2 is the coronary atherosclerosis site.

Due to atherosclerosis involves the middle layer of the blood vessels, the elastic fibers in the middle wall of the blood vessels are destroyed. Atherosclerosis is simulated by modifying Young’s modulus in middle layer of the blood vessel. When the middle layer of the blood vessels is damaged, the ability of the blood vessels to resist deformation is weakened, which can be modified by reducing the Young’s modulus. The specific Young’s modulus of the atherosclerotic site is not available and is related to the degree of coronary expansion. It is assumed that the Young’s modulus is 1/10 and 1/100 which are 0.27 and 0.027 MPa, respectively. Non-atherosclerosis sites are consistent with the normal blood vessels. The fluid-structure interaction was performed in the same manner on different models of atherosclerosis. The volume growth rate of the RCA, the LAD and the LCX under normal condition and atherosclerotic condition were compared.

### Creep Simulation

When the ability of the blood vessels to resist deformation is weakened, creep will occur with time, due to the viscoelasticity of blood vessels. A creep simulation was adopted to analyze this process. The relaxation and creep functions of the power law form have been well fitted to the experimental results of certain materials and have been widely adopted by researchers of high molecular materials. Since the temperature and stress are constant, creep rate is only related to time. The Time Hardening theory from engineering data of ANSYS WORKBENH was chosen, which is related to

(1) $$\varepsilon_{c\,r}=C_{1}\,\sigma^{C_{2}}\,t^{C_{3}}\,e^{-C_{4}/T}$$

**FIGURE 4:**
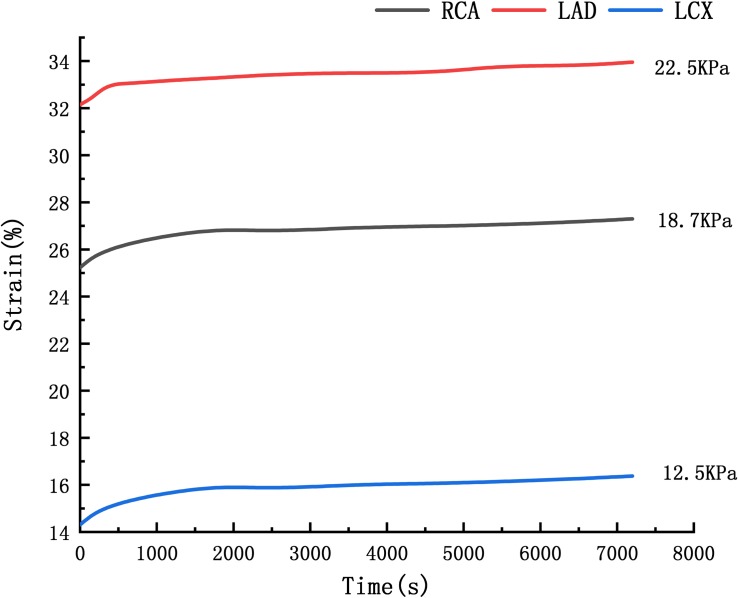
Arterial strain-time curve.

Where ε_*cr*_ is creep rate, *C*_1_–*C*_4_ are constants, σ is stress, and *T* is temperature. Li et al. (2013) performed the uniaxial creep experimental data of human cerebral arteries under 18700 and 22500 Pa. The time-strain curve at the average arterial pressure under 12500 Pa is obtained by linear interpolation of the creep experimental data under 18700 and 22500 Pa, shown in Figure 4. The C_1_–C_4_ were obtained by fitting the strain-stress curve under 12500 Pa. Fitting results are *C*_1_ = 2.35*e*^–6^, *C*_2_ = 0.6808,

*C*_3_ = 0.3273, *C*_4_ = 0. The RCA, the LAD and the LCX models are identical to the previous blood vessel model of the two-way fluid-structure interaction. The periodic load shown in Figure 3 was applied to the RCA, the LAD, and the LCX models, and the creep simulation was calculated to be 150,000 s.

## Results

### Two-Way Fluid-Structure Interaction

**FIGURE 5:**
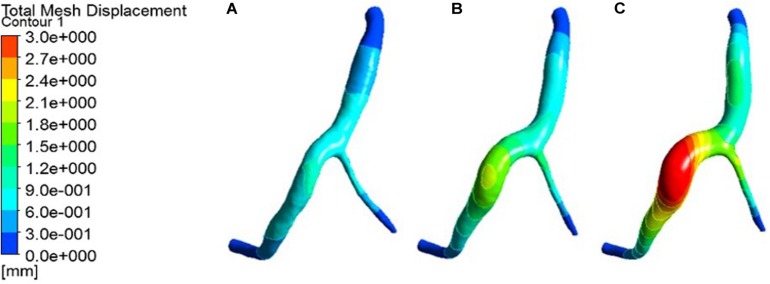
Deformation contour of the RCA in maximum time. **(A)** normal condition; **(B)** the Young’s modulus of the atherosclerotic site is 0.27MPa; **(C)** the Young’s modulus of the atherosclerotic site is 0.027 MPa.

**FIGURE 6:**
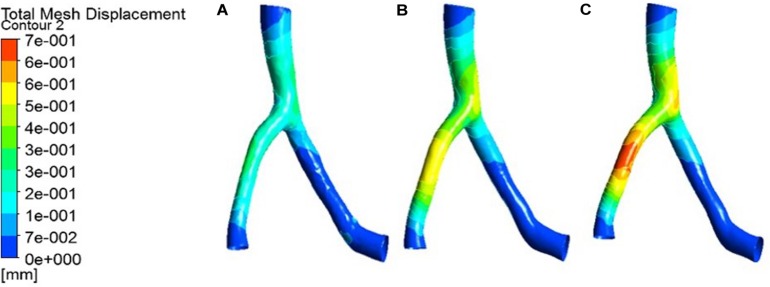
Deformation contour of the LAD in maximum time. **(A)** normal condition; **(B)** the Young’s modulus of the atherosclerotic site is 0.27MPa; **(C)** the Young’s modulus of the atherosclerotic site is 0.027 MPa.

**FIGURE 7:**
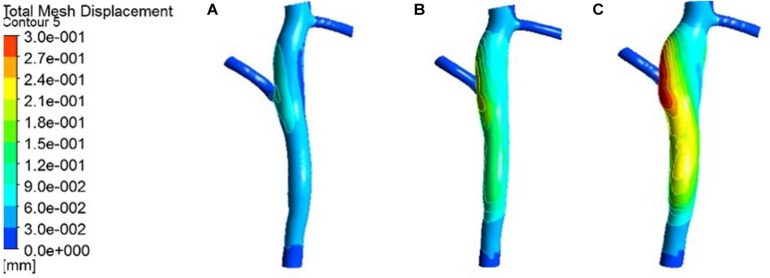
Deformation contour of the LCX in maximum time. **(A)** normal condition; **(B)** the Young’s modulus of the atherosclerotic site is 0.27MPa; **(C)** the Young’s modulus of the atherosclerotic site is 0.027 MPa.

The expansion results of the middle layer of the vessel wall under different atherosclerosis degree were calculated. The maximum deformation moment of RCA, LAD, and LCX models is 0.15. The deformation contours were derived at this time, as shown in Figures 5–7.

**FIGURE 8:**
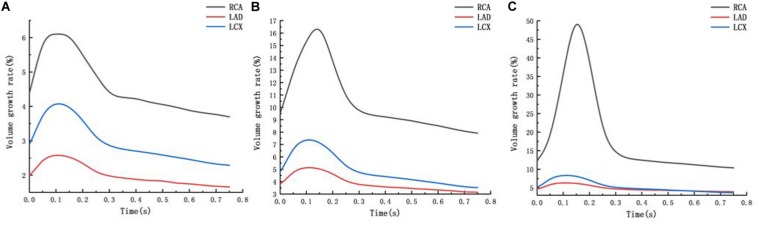
Volume growth rate curve **(A)** normal condition **(B)** the Young’s modulus of the atherosclerotic site is 0.27MPa **(C)** the Young’s modulus of the atherosclerotic site is 0.027 MPa.

From the deformation contour at the maximum time 0.15 s, it could be seen that the deformation of the RCA, the LAD and the LCX is increased after the degree of atherosclerosis is increased. The deformation of the atherosclerotic site in RCA is much larger than that in LAD and LCX. The volume growth rate curves of the atherosclerotic site in the RCA, the LAD, and the LCX could be plotted in the cardiac cycle. Volume growth rate is the ratio of increased volume to original volume after expansion, as shown in Figure 8. In a cardiac cycle, the volume growth rate of the atherosclerotic site in the RCA was larger than the LAD and LCX.

**FIGURE 9:**
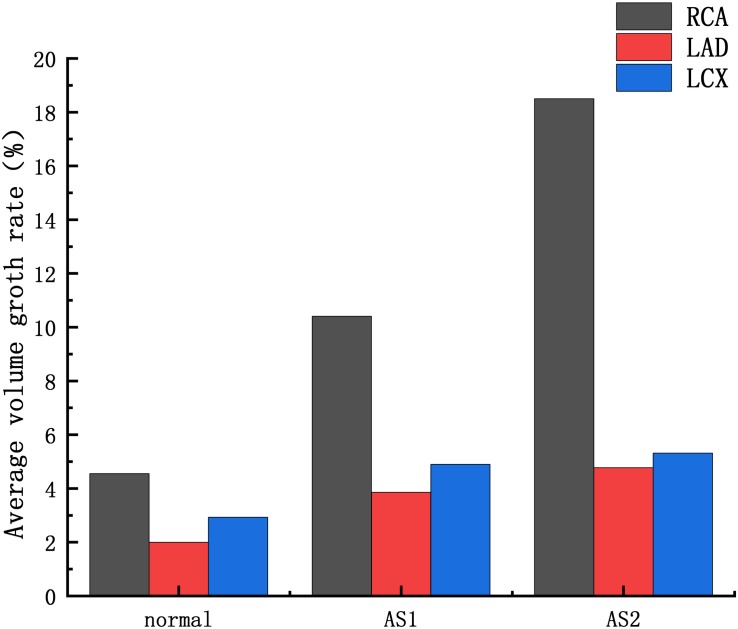
Average volume growth rate: AS1: the Young’s modulus of the atherosclerotic site is 0.27MPa; AS2: the Young’s modulus of the atherosclerotic site is 0.027 MPa.

**FIGURE 10:**
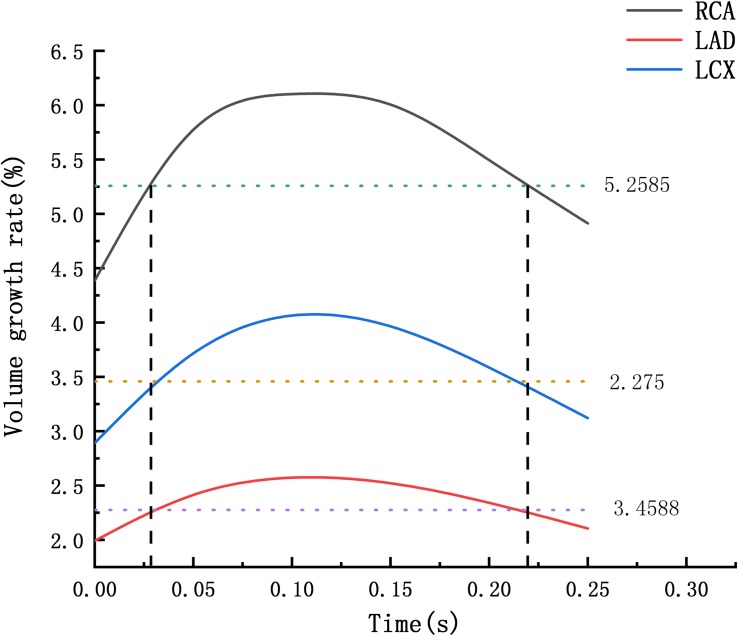
Volume growth rate in systole.

The average volume growth rates of blood vessels within a cardiac cycle were compared, as shown in. Figure 9. Under normal conditions, the average volume growth rates of the atherosclerotic site in the RCA, the LAD, and the LCX respectively were: 4.56, 2, and 2.93% in a cardiac cycle. When the middle layer of the blood vessels was damaged and the Young’s modulus was reduced to 0.27 MPa, the average volume growth rate of atherosclerotic site in the RCA, the LAD, and the LCX were 10.4, 3.86, and 4.90% respectively in a cardiac cycle. When the middle layer of the blood vessels was damaged and the Young’s modulus was reduced to 0.027 MPa, the average volume growth rate of atherosclerotic site in the RCA, the LAD, and the LCX respectively were 18.4, 4.78, and 5.32% in a cardiac cycle. Under normal condition, the volume growth rate of the RCA is 2.28 times and 1.55 times of the LAD and the LCX. After atherosclerosis, the volume growth rate of the RCA was 2.69 times and 2.12 times of the LAD and the LCX. And the volume growth rate of the RCA was 3.85 times and 3.45 times of the LAD and the LCX after further deepening of atherosclerosis. It can be seen from the volume growth curve in a cardiac cycle that vasodilation is more obvious in systole. The volume growth curves in systole (0−0.3 s) of the RCA, the LAD, and the LCX could be plotted. The average volume growth rates of the RCA, the LAD, and the LCX in systole respectively were 5.26, 2.28, and 3.46%. These were shown in Figure 10. The auxiliary lines from the intersection of average line of the RCA and volume growth curve of the RCA to *X* axis were plotted. It can be seen that the time when volume growth rate of the RCA is higher than the average was longer than the LAD and the LCX, which respectively were 0.027−0.221 s, 0.052−0.22 s, and 0.033−0.212 s.

### Creep Simulation

**FIGURE 11:**
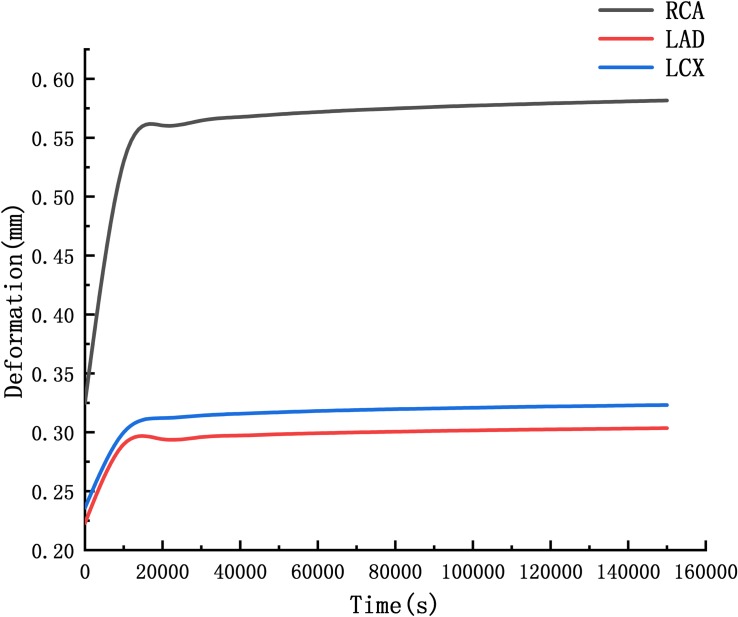
Curve of deformation.

The deformation curves of the RCA, LAD, and LCX were obtained by creep simulation. As shown in Figure 11, it could be seen that the deformation of the RCA was larger than the LAD and the LCX.

Deformation functions of the RCA, LAD, and LCX were obtained by fitting the curve in Figure 11.

D=R⁢C⁢A4.67et-4018410.

D=L⁢A⁢D2.52et-4015610.

D=L⁢C⁢X2.63et-4017280.

Through the function we can predict that after 10 years, the RCA is 2.06 times the original, the LAD and LCX are 1.53 times and 1.56 times. It indicated that the RCA is more likely to form CAA than the LAD and LCX.

## Discussion

At present, the study of coronary aneurysms is more common in a few clinical reports, and its hemodynamic mechanism research is still insufficient. From clinical statistics, coronary aneurysms can be found to be highly focal and easily occur in the RCA. The formation of CAA is a complex physiological process. The Fluid-structure Interaction numerical simulations were performed to compare the expansion ability of the RCA, the LAD and LCX. The Creep simulations were performed to predict the formation of CAA. These simulations attempt to explain the hemodynamic mechanism of coronary artery aneurysm easily occurring on RCA The average volume growth rate of the RCA under normal condition and atherosclerosis both larger than the LAD and the LCX, shown in Figure 9. In the Fluid-structure Interaction numerical simulations, there is almost no difference between the material of the blood vessels and the pressure in the vessels. It can be considered that the structural difference makes the RCA prone to expand. Coronary diameter statistics were performed on 167 subjects. The average diameter of the RCA, the LAD and the LCX respectively are 2.95 ± 060, 2.14 ± 0.43, and 2.26 ± 0.41 mm. It can be seen that the average diameter of the RCA is greater than the LAD and the LCX. The larger average diameter of the RCA leads it prone to expand. From Figure 9, it can be seen that under normal condition, the volume growth rate of the RCA is 2.28 times and 1.55 times of the LAD and the LCX. After atherosclerosis, the volume growth rate of the RCA was 2.69 times and 2.12 times of the LAD and the LCX. And the volume growth rate of the RCA was 3.85 times and 3.45 times of the LAD and the LCX after further deepening of atherosclerosis. It indicates that the larger expansion is magnified under atherosclerosis condition with destroyed vessel elasticity. As well as, it can be seen that the time above average volume growth rate in systole of the RCA, LAD, and LCX respectively were 0.194, 0.168, and 0.179 s, shown in Figure 10. In Creep simulations, this accumulation of the time difference leads the RCA reaching to 2.06 times, while the LAD and the LCX respectively were 1.53 times and 1.56 times after 10 years. So it can be considered that the RCA is more prone to aneurysms originated from the larger expansion of the RCA under normal physiological condition, and the larger expansion is magnified under atherosclerosis condition with destroyed vessel elasticity, and further magnified during the time accumulated viscoelastic creep to develop to aneurysm eventually.

There are some limitations in our study. The formation of CAA is a complex physiological process. Many physiological factors have not been considered. Human blood vessels are anisotropic, and stress-strain are not a simple linear relationship. The linear elastic material given in the article is indeed not as suitable as the superelastic material in reflecting the deformation of blood vessels. Due to the limitation of calculation cost, the simulation time of the creep process is 150,000 s. We can only give the trend of coronary artery dilation, but cannot really simulate the formation of CAA. The differences in the location and structure of atherosclerosis were not considered in this study. With the expansion of the blood vessel, wall shear stress at the site of atherosclerosis decreases, which may lead to intimal hyperplasia, deepening of atherosclerosis, and affecting coronary dilation. However, the potential interaction between low wall shear stress and coronary dilation has not yet been clarified, and further work is needed. These problems will continue to be explored in future research.

## Conclusion

In this article, numerical simulations show that RCA is more likely to expand to form CAA, which is consistent with clinical statistics. The RCA is more prone to aneurysms originated from the larger expansion of the RCA under normal physiological condition, and the larger expansion is magnified under atherosclerosis condition with destroyed vessel elasticity, and further magnified during the time accumulated viscoelastic creep to develop to aneurysm eventually.

## Data Availability Statement

The raw data supporting the conclusions of this article will be made available by the authors, without undue reservation, to any qualified researcher.

## Author Contributions

DW participated in all the work and wrote the manuscript. YL contributed conception and design of the study. SW organized the database. BM and BL performed the statistical analysis. CJ and YF collected relevant documents. GL provided guidance on research methods and language. All authors contributed to manuscript revision, read and approved the submitted version.

## Conflict of Interest

The authors declare that the research was conducted in the absence of any commercial or financial relationships that could be construed as a potential conflict of interest.

## References

[B1] Anabtawi I. N. De Leon J. A. (1974). Arteriosclerotic aneurysms of the coronary arteries. *J. Thor. Cardiovasc. Surg.* 68 226–228. 4546268

[B2] Baman T. S. Cole J. H. Devireddy C. M. Sperling L. S. (2004). Risk factors and outcomes in patients with coronary artery aneurysms. *Am. J. Cardiol.* 93 1549–1551. 15194034 10.1016/j.amjcard.2004.03.011

[B3] Dahhan A. (2015). Coronary artery ectasia in atherosclerotic coronary artery disease, inflammatory disorders, and sickle cell disease. *Cardiovasc. Ther.* 33 79–88. 10.1111/1755-5922.12106 25677643

[B4] Demopoulos V. P. Olympios C. D. Fakiolas C. N. Pissimissis E. G. Economides N. M. Adamopoulou E. (1997). The natural history of aneurysmal coronary artery disease. *Heart* 78 136–141. 9326986 10.1136/hrt.78.2.136 PMC484892

[B5] Gao F. Guo Z. Sakamoto M. Matsuzawa T. (2006). Fluid-structure interaction within a layered aortic arch model. *J. Biol. Phys.* 32 435–454. 10.1007/s10867-006-9027-7 19669449 PMC2651537

[B6] Jariwala P. Padmakumar E. A. Krishnaprasad A. R. (2018). Acutecoronary syndrome secondary to coronary artery aneurysms: case reports and review. *Ihj Cardiovasc. Case Rep.* 2 85–90.

[B7] Kim H. J. Vignon-Clementel I. E. Coogan J. S. Figueroa C. A. Jansen K. E. Taylor C. A. (2010). Patient-specific modeling of blood flow and pressure in human coronary arteries. *Ann. Biomed. Eng.* 38 3195–3209. 10.1007/s10439-010-0083-6 20559732

[B8] Li B. Wang W. Mao B. Liu Y. (2018). A method to personalize the lumped parameter model of coronary artery. *Int. J. Compu. Methods* 2018:184 2004.

[B9] Li D. Y. Xu D. H. Li P. Wei J. Yang K. Zhao C. H. (2013). Viscoelastic evaluation of fetal umbilical vein for reconstruction of middle cerebral artery. *Chin. Neuroregen. Res* 32 3055–3062. 10.3969/j.issn.1673-5374.2013.32.009 25206626 PMC4146204

[B10] Li N. Guo T. (2017). “A multi-layer finite element model based on anisotropic hyperelastic of coronary artery and it’s application,” in *Proceedings of the International Conference on Bioinformatics and Biomedical Engineering (iCBBE2011)*, Kyoto.

[B11] Maier A. Gee M. W. Reeps C. Eckstein H. H. Wall W. A. (2010). Impact of calcifications on patient-specific wall stress analysis of abdominal aortic aneurysms. *Biomech. Model. Mechanobiol.* 9 511–521. 10.1007/s10237-010-0191-0 20143120

[B12] Pahlavan P. S. Niroomand F. (2006). Coronary artery aneurysm: a review. *Clin. Cardiol.* 29 439–443. 17063947 10.1002/clc.4960291005 PMC6654377

[B13] Pan Z. X. Wang Y. T. Dou K. F. You S. J. Song W. H. (2014). Clinical characteristics of 669 hospitalized patient with Coronary expansion (CAE). *Chin. J. Mol. Cardiol.* 14 1120–1123.

[B14] Stergiopulos N. Meister J. J. Westerhof N. (1996). Determinants of stroke volume and systolic and diastolic aortic pressure. *Am. J. Physiol. Heart Circul. Physiol.* 270 H2050–H2059. 10.1152/ajpheart.1996.270.6.H2050 8764256

[B15] Swaye P. S. Fisher L. D. Litwin P. Vignola P. A. Judkins M. P. Kempet H. G. (1983). Aneurysmal coronary artery disease. *Circulation* 67:134. 6847792 10.1161/01.cir.67.1.134

[B16] Syed M. M. (1997). Coronary artery aneurysm: a review. *Prog. Cardiovasc. Dis.* 40 77–84. 9247557 10.1016/s0033-0620(97)80024-2

[B17] Taylor C. A. Fonte T. A. Min J. K. (2013). Computational fluid dynamics applied to cardiac computed tomography for noninvasive quantification of fractional flow reserve: scientific basis. *J. Am. Coll. Cardiol.* 61 2233–2241. 23562923 10.1016/j.jacc.2012.11.083

[B18] Watanabe F. M. Matsuzawa T. (2006). Stress analysis in a layered aortic arch model under pulsatile blood flow. *Biomed. Eng. Online* 5:25. 16630365 10.1186/1475-925X-5-25 PMC1513233

[B19] Zhao X. Liu Y. Ding J. Bai F. Ren X. Ma L. (2014). Numerical study of bidirectional glenn with unilateral pulmonary artery stenosis. *J. Mech. Med. Biol.* 14:1450056.

[B20] Zhao X. Liu Y. Li L. Wang W. Xie J. Zhao Z. (2016). Hemodynamics of the string phenomenon in the internal thoracic artery grafted to the left anterior descending artery with moderate stenosis. *J. Biomech.* 49 983–991. 26972762 10.1016/j.jbiomech.2015.11.044

